# Metallothionein 2A genetic polymorphisms and risk of ductal breast cancer

**DOI:** 10.1007/s10238-012-0215-4

**Published:** 2012-10-06

**Authors:** Anna Krześlak, Ewa Forma, Paweł Jóźwiak, Agnieszka Szymczyk, Beata Smolarz, Hanna Romanowicz-Makowska, Waldemar Różański, Magdalena Bryś

**Affiliations:** 1 Department of Cytobiochemistry, University of Łódź, Pomorska 141/143, 90-236 Lodz, Poland; 2 Department of Clinical Pathomorphology, Polish Mother’s Memorial Hospital Research Institute, Rzgowska 281/289, 93-338 Lodz, Poland; 3 2nd Department of Urology, Medical University of Łódź, Pabianicka 62, 93-513 Lodz, Poland

**Keywords:** Metallothionein 2A, Breast cancer, SNP

## Abstract

Metallothioneins (MTs) are a family of metal binding proteins that play an important role in cellular processes such as proliferation and apoptosis. Metallothionein 2A is the most expressed MT isoform in the breast cells. A number of studies have demonstrated increased MT2A expression in various human tumors, including breast cancer. We carried out an association study to examine whether MT2A gene polymorphisms are associated with risk of breast cancer. Information on lifestyle risk factors was collected via a self-administered questionnaire. Genotyping was conducted using polymerase chain reaction–restriction fragment length polymorphism technique. Three single nucleotide polymorphisms (SNP) rs28366003, rs1610216 and rs10636 were genotyped in 534 breast cancer cases and 556 population controls. One SNP in MT2A (rs28366003) showed a positive association with breast cancer. Compared with homozygous common allele carriers, heterozygous for the G variant [odds ratio (OR) = 1.92, 95 % confidence interval (CI):1.28–2.81, *p* trend <0.01; the OR assuming a dominant model 1.93 (95 % CI: 1.29–2.89, *p*_dominant_ <0.02) after adjustment for age, family history, smoking status, BMI, menarche, parity, menopausal status and use of contraceptive and menopausal hormones] had a significantly increased risk of breast cancer in Polish population, as well as women with haplotypes, including variant allele of rs28366003 SNP (OR = 1.58, CI: 0.41–6.33, *p* global = 0.03). Our data suggest that the rs28366003 SNP in MT2A is associated with risk of breast cancer in Polish population.

## Introduction

Metallothioneins (MTs) are a family of proteins with low molecular mass (6–7 kDa), high content of cysteine and complete absence of aromatic amino acids [1–3]. MTs bind to biologically essential metals like Zn and Cu and can participate in regulation of these metals homeostasis. Moreover, MTs absorb the heavy metals such as Cd, Pb, Hg and As and assist with their transportation and extraction [1–4]. Apart from their role in protection of tissue against heavy metals, MTs can act as potent antioxidants against oxidative damages [1–4]. They are known to participate also in cell proliferation and apoptosis which are very important processes in carcinogenesis [5–7]. In human, MTs are encoded by a family of genes located on chromosome 16q13 consisting of ten functional MT isoforms. The encoded proteins are subdivided into four groups: MT1, MT2, MT3 and MT4 proteins. Human MT isoforms have tissue-specific expression patterns [3]. MTs expression increases in response to various inducers such as metals, interleukins, interferon, tumor necrosis factor alpha and glucocorticoid hormones [8].

A number of studies have demonstrated increased expression of MT1 and MT2 mRNA and protein in various human tumors such as kidney, lung, nasopharynx, ovary, prostate, salivary gland, testes, urinary bladder, cervical, endometrial, skin and pancreatic cancers as well as melanoma [7, 9, 10]. In some cases, high expression of MT1 and MT2 correlates with tumor grade/stage, chemotherapy resistance and poor prognosis. However, MT1/2 can also be down-regulated in certain tumors, for example, hepatocellular carcinoma and liver adenocarcinoma [7].

MT2A is the most expressed isoform in the breast cells. The MT2A mRNA expression is positively correlated with histological grade [7]. Histological grade 3 tumors were observed to have a higher expression of MT2A mRNA than grade 1 and 2 tumors. Immunohistochemical studies have shown that cytoplasmic and/or nuclear MT1/2 protein expression is associated with tumor grade, increased recurrence rate and poor survival in the highly malignant invasive ductal breast carcinomas [5, 7, 9, 10]. Some studies have shown an inverse correlation between MTs expression and progesterone and estrogen receptors positivity [11]. Based on correlation of MT expression and clinicopathological parameters of breast cancer showed in many reports, Bay et al. [12] suggested that metallothionein is a promising prognostic biomarker in breast cancer.

Taking into account the potential role of MT2A in breast carcinogenesis, we have analyzed an association between breast cancer and three known single nucleotide polymorphisms (SNPs) of MT2A gene. We selected SNPs that could potentially affect gene expression. The SNPs analyzed by us in this study concerned A/G transitions in promoter region at loci −5 and −209 (rs28366003 and rs1610216, respectively) and C/G transition in 3’UTR region at locus +838 (rs10636). Therefore, we comprehensively evaluated the association of this SNP with breast cancer risk in a Polish population-based case–control study.

## Materials and methods

### Study population

This case–control study involved 534 women with invasive ductal breast cancer (age range 34–81, mean age 54.76 ± 7.35) recruited between May 2003 and November 2010. The patients had a confirmed diagnosis of ductal breast cancer based on histopathological evaluation and were under treatment at the Polish Mothers Memorial Hospital Research Institute, Lodz, Poland. None of the recruited patients received preoperative chemo- or radiotherapy. Patients with recurrence of breast cancer and patients who had previously diagnosed with other types of primary cancers (excluding skin cancer) were excluded.

A group of 556 healthy Polish individuals were collected from the Polish Mothers Memorial Hospital Research Institute from periodic health checkups and used as reference. They were non-related women who have never been diagnosed with breast tumors or other tumors and were randomly selected and matched to the cases on 10 years age groups (age range 34–83, mean age 51.27 ± 11.18). We enrolled only Caucasian women born and living in central Poland (Łódź region). We queried the cancer registry database every 2 months and identified all histopathologically confirmed incident primary breast cancer cases reported within 4 months of diagnosis preceding the recruitment. Informed consent was obtained from patients and controls, and the Ethical Committee approved the study.

### Lifestyle risk factors

Study participants were interviewed using questionnaire that included socio-demographic, health related information, smoking status, menstrual and reproductive histories and exogenous hormone use. A positive family history of breast cancer was defined as reporting of breast cancer in one or more first-degree relatives. Body mass index (BMI) was calculated from current weight in kilograms divided by height in meter square (measurements were extracted from patient’s medical record). Smoking was grouped into “never,” “former” and “current” based on self-reported usage. Participants who reported smoking at least 100 cigarettes in their lifetime and who, at the time of survey, smoked either every day or some days were defined as current smoker. Eight participants reported smoking at least 100 cigarettes in their lifetime but had not been smoking for 1–2 months. They were classified as current smoker. Participants who reported smoking at least 100 cigarettes in their lifetime and who had not been smoking for at least 3 months were defined as former smoker. Participants who reported never having smoked 100 cigarettes were defined as never smoker. Natural age at menopause was defined as the age at the last menstrual period, which can only be defined retrospectively after at least 12 consecutive months of amenorrhea. This last menstrual period should not be induced by surgery or other obvious causes, such as irradiation or hormone therapy. None of the women involved in the study had undergone a hysterectomy and oophorectomy. Regular drug use was defined as self-report use of oral contraceptives or menopausal hormones for 6 months or longer. For any missing survey data, patients were subsequently queried which lead to complete responses by all participants.

### Questionnaire and the matching techniques

An analysis of the computerized medical records identified 916 ductal breast cancer patients in the age group 30–85 years. From the same hospital registry of women undergoing periodic health checkups, a control population of 1,082 individuals in the same age group (30–85 years) was randomly selected. A postal questionnaire was sent to both the cases and controls. The response rate in the survey for the ductal breast cancer patients was 58.3 % and for the controls 56.3 % thus leaving a group of *n* = 534 cases and a population of *n* = 609 controls. Finally, the study group was 534 cases and 556 controls.

### Venipuncture, blood sample collection and genotyping

The larger median cubital, basilic or cephalic veins were selected and the tourniquet was applied 3–4 inches above the collection site. Next, the puncture site was cleaned by the 70 % alcohol pad, 21G needle was inserted into a vein, and blood started to flow to collection tube with EDTA as an anticoagulant (lavender top). After blood collection, tube was inverted 8–10 times and placed in refrigerator at −20 °C.

Blood samples obtained from the participants were stored at −20 °C within 2 h of removal. DNA was isolated by standard method using proteinase K digestion, phenol chloroform extraction and ethanol precipitation.

The quantity and purity of the DNA extracts were assessed by spectrophotometry using the Helios Alpha UV–vis spectrophotometer system (Thermo Fisher Scientific Inc.). The absorbances at 230, 260 and 280 nm were measured in 2 μl of each sample. Concentration of DNA was estimated by spectrophotometric quantification at 260 nm. The overall purity was assessed by calculating the absorbance ratios 260/280 and 260/230. High values for both ratio (260/280 > 1.8, 260/230 > 2) are commonly accepted as good indicators for pure DNA.

PCR reactions were performed, using Perkin–Elmer DNA Thermal cycler 480, in a total volume of 50 μl to amplify MT2A −5A/G, −209A/G and +838C/G. The reaction mixtures consisted of 100 ng of genomic DNA and the following set of primers: 10 μM of MT2A −5A/G primers (5′-GGGCCGCCTTCAGGGAACTG-3′ and 5′-GGACTTGGAGGAGGCGTGGT-3′) MT2A −209A/G primers (5′-GGCTCAGGTTCGAGTACAGG-3′ and 5′-AAGTCACTTGCGGCTCCA-3′) or of MT2A +838 primers (5′-CCGCTCCCAGATGTAAAGAA-3′ and 5′-GGCATATAAAGAAAACCAGAGACA-3′). The DNA samples were amplified in the presence of 200 μmol dNTPs, 10 % dimethylsulfoxide, 1× Taq polymerase buffer, 1.5 mM MgCl_2_ and 0.5 U AmpliTaq Gold (Applied Biosystems, Foster, CA). The PCR condition for MT2A −5A/G comprised an initiation denaturation step at 94 °C for 4 min, followed by 30 cycles of 96 °C for 1 min, 61 °C for 1 min, 72 °C for 1 min and final extension step at 72 °C for 10 min. MT2A −209A/G was amplified 35 cycles at an annealing temperature of 62 °C for 1 min, and extension at 72 °C for 10 min. The conditions for MT2A +838C/G was 30 cycles at 61 °C annealing temperature for 1 min and extension at 72 °C for 10 min. Subsequently, RFLP analysis was performed on 20 microliters each of the respective PCR products by subjecting them to the following restriction enzymes: at a 5 U concentration: *Bsg* I (at 37 °C for 3 h) for MT2A −5A/G and *Sma* I (at 25 °C for 3 h) for MT2A −209A/G (both from New England Biolabs, UK), and *Mae* III (Roche Molecular Systems, Branchburg, USA) for MT2A +838C/G with 3 U concentration and overnight incubation at 55 °C [13–15].

In the case of MT2A −5A/G, the lengths of fragments obtained by digestion of each 185-bp fragment by *Bsg* I were 144 and 41 bp for the A/A genotype, 185, 144 and 41 bp for the A/G type and 185 bp for the G/G genotype. For MT2A −209A/G, after digestion by *Sma* I of the PCR products of 246 bp fragment for A/A homozygotes, 131 and 115 bp for G/G homozygotes and all three fragments for heterozygotes A/G were registered. Analogously, in the case of MT2A +838C/G, after digestion by *Mae* III of the PCR products of 157-bp fragment for A/A homozygotes, 95 and 62 bp for G/G homozygotes and 157, 95 and 62 bp for heterozygotes A/G were observed.

The products were analyzed by electrophoresis on 3 % agarose gel and ethidium bromide-stained. Positive and negative controls were included in each gel. Quality control was ensured by including a random 5 % of the samples as duplicates.

### Statistical data analysis

Genotype distributions were evaluated for agreement with Hardy–Weinberg equilibrium by the Chi-square test. All genotype distributions of MT2A fit Hardy–Weinberg equilibrium. Unconditional multiple logistic regression models were used to calculate odds ratios (ORs) and 95 % confidence intervals (CIs) for the association of genotype with breast cancer risk. Genotype data were analyzed with the homozygote of the common allele as the reference group. Variants of homozygotes and heterozygotes were combined to evaluate the dominant effect. For each SNP, trend tests were conducted by assigning the values 1, 2 and 3 to homozygous wild type, heterozygous and homozygous variant genotypes, respectively, and by adding these scores as a continuous variable in logistic regression model.

The haplotype effects of the polymorphisms on breast cancer risk were analyzed using the Chaplin 1.2 (genetics.emory.edu) and THESIAS software (www.genecanvas.org). All haplotypes were examined simultaneously in regression models with the most common haplotype as the reference. Haplotypes were evaluated for association with breast cancer in unadjusted and adjusted logistic regression models as done for the individual SNPs. All multivariate models were adjusted for age, family history, obesity, smoking status, parity, menopausal status and use of contraceptive and menopausal hormones. Reported *p* values were two sided. Probabilities were considered significant whenever *p* value was lower than 0.05. All analyses were completed using STATA software (version 11.0 StataCorp., Texas, USA).

## Results and discussion

The distributions of socio-demographic characteristics and lifestyle risk factors are shown in Table 1. Our sample is as a whole Caucasian and closely mirrors the Polish population. The cases were slightly older (54.76 ± 7.35 vs. 51.27 ± 11.18 years for controls), more likely to use oral contraceptives (64.4 vs. 50.1 % for controls), more likely to be a current smoker (37.1 vs. 26.2 % for controls) and more likely to have a BMI greater or equal than 30 (39.9 vs. 28.9 % for controls).

**Table 1:** Selected baseline characteristics of breast cancer cases and controls with questionnaire data

	Cases (*n* (%))	Controls (*n* (%))	*p*
*Age (years)*			
35–44	123 (23.0)	189 (34.0)	
45–54	133 (25.0)	139 (25.0)	
55–64	150 (28.1)	122 (21.9)	
65–74	96 (18.0)	89 (16.0)	
75–84	32 (5.9)	17 (3.1)	<0.001
*Family history of breast cancer* ^*a*^			
Yes	64 (11.9)	50 (9.0)	
No	470 (88.1)	506 (91.0)	0.11
*Obesity (BMI* ≥ *30* *kg/m2)* ^*b*^			
Yes	213 (39.9)	161 (28.9)	
No	321 (60.1)	395 (71.1)	<0.001
*Smoking status*			
Never	96 (18.0)	155 (27.9)	
Former	240 (44.9)	255 (45.9)	
Current	198 (37.1)	146 (26.2)	<0.001
*Menarche (years)*			
10	11 (2.1)	0 (0.0)	
11	101 (18.9)	106 (19.2)	
12	171 (32.1)	200 (35.9)	
13	144 (26.9)	167 (30.0)	
14	91 (17.1)	72 (12.9)	
≥15	16 (2.9)	11 (2.0)	<0.01
*Used oral contraceptives* ^*c*^			
Yes	344 (64.4)	283 (50.1)	
No	190 (35.6)	273 (49.9)	<0.0001
*Parity*			
Nulliparous	114 (21.3)	128 (23.0)	
1	125 (23.4)	144 (25.9)	
2	140 (26.2)	156 (28.0)	
3	98 (18.3)	94 (16.9)	
≥4	57 (10.8)	34 (6.2)	0.07
Age at menopause(years)^d^	50.1 ± 4.3	49.8 ± 4.6	0.23
*Use of menopausal hormones* ^*e*^			
Never	308 (57.7)	384 (69.1)	
Estrogen	144 (27.0)	94 (16.9)	
Progestin	32 (6.0)	23 (4.1)	
Combined	50 (9.3)	55 (9.9)	<0.001

^a^Family history defined as self-reporting of at least one first-degree relative with known breast cancer

^b^Body mass index (BMI) was calculated as current weight in kilograms divided by height in meter square (according to WHO classification)

^c^Regular drug use was defined as self-report use of oral contraceptives for 6 months or longer

^d^Natural age at menopause was defined as the age at the last menstrual period, which can only be defined retrospectively after at least 12 consecutive months of amenorrhea. This last menstrual period should not be induced by surgery or other obvious causes, such as irradiation or hormone therapy

^e^Regular drug use was defined as self-report use of menopausal hormones for 6 months or longer

Genotype and allele distributions for MT2A polymorphisms in 534 breast cancer patients and 556 control subjects are summarized in Table 2. Approximately, 95 % of the PCR–RFLP reactions were successful (4.7 % cases and 5.3 controls had unsuccessful PCR reactions). One SNP in MT2A (rs28366003) showed a positive association with breast cancer. Having one copy of the risk allele (G) conferred an estimated 90 % increase in breast cancer in the model adjusted for age, family history, smoking status, BMI, menarche, parity, menopausal status and use of contraceptive and menopausal hormones (OR = 1.94, 95 % CI: 1.31–2.90, *p*_dominant_ <0.02). Homozygous variant allele carriers were not present in healthy controls. No other SNP was statistically significantly associated with breast cancer. Four haplotypes were estimated to have a population frequency of at least 5 %. One haplotype GAG, including variant allele of rs28366003 SNP, showed evidence of a statistically significantly increased risk of breast cancer in Polish population, with an approximately 50 % increase in risk for (OR = 1.58, CI: 0.41–6.33, *p* global = 0.03) (Table 3).

**Table 2:** Associations between MT2A SNPs and breast cancer risk

SNP genotype	Cases (%)/controls (%)	OR (95 % CI)^a^	*p*	OR (95 % CI)^b^	*p*
*rs28366003 (SNP1)*					
AA	465 (87.1)/516 (92.8)	1.00 (reference)		1.00 (reference)	
AG	66 (12.3)/40 (7.2)	1.83 (1.21–2.77)	–	1.92 (1.28–2.81)	
GG	3 (0.6)/0 (0.0)	–		–	
*p* trend^c^		<0.001		<0.01	
AG or GG vs. AA^d^	69 (12.9)/40 (7.2)	1.91 (1.27–2.88)	<0.01	1.93 (1.29–2.89)	<0.02
AG or AA vs. GG^e^	531 (99.4)/556 (100)	–	–	–	–
*rs1610216 (SNP2)*					
AA	408 (76.4)/402 (72.3)	1.00 (reference)		1.00 (reference)	
AG	125 (23.4)/153 (27.5)	0.80 (0.61–1.06)		0.81 (0.63–1.09)	
GG	1 (0.2)/1 (0.2)	0.98 (0.06–15.83)		1.06 (0.09–16.72)	
*p* trend^c^		0.13		0.73	
AG or GG vs. AA^d^	126 (23.6)/154 (27.7)	0.81 (0.61–1.06)	0.12	0.82 (0.64–1.11)	0.24
AG or AA vs. GG^e^	533 (99.8)/555 (99.8)	1.04 (0.06–16.69)	0.98	0.97 (0.07–15.41)	0.87
*rs10636 (SNP3)*					
GG	305 (57.1)/280 (50.3)	1.00 (reference)		1.00 (reference)	
GC	205 (38.4)/253 (45.5)	0.74 (0.58–0.95)		0.69 (0.52–0.94)	
CC	24 (4.5)/23 (4.2)	0.96 (0.53–1.74)		0.91 (0.64–1.71)	
*p* trend^c^		0.07		0.17	
CG or CC vs. GG^d^	510 (95.5)/504 (90.6)	0.76 (0.60–0.97)	0.02	0.73 (0.57–0.96)	0.06
CG or GG vs. CC^e^	229 (42.9)/301 (54.1)	1.09 (0.61–1.96)	0.07	1.11 (0.64–2.03)	0.94

^a^Crude

^b^Adjusted for age, family history, smoking status, BMI, menarche, parity, menopausal status and use of contraceptive and menopausal hormones

^c^Testing additive genetic model (Cochran–Armitage test for trend)

^d^Testing dominant genetic model

^e^Testing recessive genetic model

**Table 3:** Associations between MT2A haplotypes and breast cancer risk

Haplotypes (SNP1–SNP3)	Cases (%)/controls (%)	OR (95 % CI)^a^	OR (95 % CI)^b^
A-A-G	167 (31.27)/184 (33.09)	1.00 (reference)	1.00 (reference)
G-A-G	142 (26.58)/121 (21.77)	1.54 (0.38–6.28)	1.58 (0.41–6.30)
A-A-C	118 (22.10)/134 (24.10)	0.84 (0.37–1.65)	0.83 (0.36–1.67)
A-G-C	106 (19.85)/117 (21.04)	0.82 (0.42–2.19	0.81 (0.41–2.08)

^a^Crude

^b^Adjusted for age, family history, smoking status, BMI, menarche, parity, menopausal status and use of contraceptive and menopausal hormones

In this breast cancer case–control study in a Polish population, we examined the association of three single nucleotide polymorphisms of MT2A gene with the risk of breast cancer. The SNPs analyzed by us concerned A/G transition in the promoter region at loci −5 and −209 (rs28366003 and rs1610216, respectively) and C/G transition in the untranslated region at locus +838 (rs10636). Our results suggest that one of three SNPs (rs28366003) influences significantly susceptibility of breast cancer.

MT2A −5A/G single nucleotide polymorphism (rs28366003) is located at the core promoter region of MT2A gene between the TATA box and the site of initiation of transcription. This SNP is an A/G substitution located in the center of the consensus sequence TGCACTC. This conversion in turn may reduce the binding, to the core promoter region, of a nuclear protein that is involved in MT2A gene basic transcription [13].

In the literature, there are a few studies concerning rs1610216, rs28366003r and rs10636 polymorphisms of *MT2A*. The −5A/G polymorphism has been studied in Japanese and Turkish populations and genotype frequencies of AA, AG and GG are 82, 17 and 0,9 % for Japanese and 87, 12,3 and 0,7 % for Turkish [13, 16]. McElroy et al. [17] determined the frequency of the A and G alleles at rs28366003 in the promoter region of *MT2A* in White and Black females in the Midwestern United States. The frequency of the G allele was 1.1 % for Blacks and 6.4 % for Whites. Data demonstrated that the G allele is not common in both the Midwestern US Black and White female population and is less frequent than that reported for an Asian population. In our study regarding −5 A/G polymorphism, the allelic frequencies of the A/A, A/G, and G/G genotypes in the control group (healthy subjects) were 92.8, 7.2 and 0 %, respectively. Thus, similarly to the results obtained in Midwestern US, female population frequency of G allele was rather low in Polish women population.

The rs28366003 polymorphism was extensively studied in Turkish population in relation to cadmium and other metals levels in tissues and blood [18–20]. The other metallothionein 2A gene polymorphisms have been studied for their associations with risk for a variety of diseases. Giacconi et al. [14] reported a significant relationship between −209A/G *MT2A* polymorphism, diabetes type II and atherosclerosis. The positive association between +838 C/G *MT2A* polymorphism and carotid artery stenosis in elderly people was also found [15]. Yang et al. [21] showed that rs10636 was significantly associated with incidence of type 2 diabetes mellitus with neuropathy in Chinese population.

The role of MT in carcinogenesis and progression of breast cancer is not fully understood but it is suggested that metallothionein may play a role in cell proliferation, apoptosis and differentiation which are very important processes in carcinogenesis. The MT2A expression in breast cancer tissue correlates with increased proliferation indicated by Ki-67 immunopositivity [22]. Moreover, it was demonstrated that overexpression of MT2A in breast MCF-7 cells resulted in a twofold increase in cell multiplication [23]. Lim et al. [24] suggested that MT2A could plausibly modulate cell cycle progression from G1 to S phase via the ATM/Chk2/cdc25A pathway. Antisense down-regulation of MT2A was associated with increased apoptosis [11]. It is suggested that metallothionein participates in the regulation of apoptosis by inhibiting cell death and improving cell survival in periods of stress due to its ability to prevent oxidative damage and ability to interact with different apoptotic signal mechanisms [25, 26]. Metallothionein may also play a role in cancer progression. Kim et al. [27] have shown that metallothionein overexpression in MDA-MB-231 cells increased the expression of metalloproteinase-9. They suggest that up-regulation of MMP-9 is one of the mechanisms by which MT2A promotes invasion of breast cancer cells.

Taking into account these molecular and cellular studies, it seemed to be very likely that genetic polymorphisms in the MT genes may affect susceptibility to breast cancer. In the literature, there is only one study of *MT2A* polymorphism concerning breast cancer patients. Seibold et al. [28] have found the association of *MT2A* polymorphisms with postmenopausal breast cancer risk. The two significant SNPs (rs1008766 and rs1580833) analyzed by them are located in flanking regions of the gene. Our results suggest that another SNP (rs28366003) might also be associated with breast cancer risk.

There are several limitations of our study. One of them is the small sample size and the small number of homozygote individuals with rare alleles which may affect odds ratio estimates. Our results are limited to Caucasian women born and living in central Poland. Further studies are needed in other populations. Moreover, all cases used in this study are invasive ductal breast cancer, the most common type of invasive breast cancer. Our findings may not generalize to less frequently diagnosed invasive breast cancer subtypes.

While confirmation of our results in other populations will be necessary, the results of this study are consistent with other studies suggesting the potential role of MT2A gene in breast carcinogenesis. Since the −5 position is located in the promoter region, it is possible that the substitution A/G produces allele-specific MT2A gene expression. Our future research will address just this issue in relation to the concentration of metals in breast cancer tissue. In summary, we found that in Polish population, the −5A/G (rs28366003) polymorphism in the MT2A gene may play role in susceptibility to breast cancer.
